# Does Early Concordant Antibiotic Treatment Reduce Mortality Among Hospitalized Patients with Carbapenem-Resistant *Acinetobacter baumannii* Bacteremia? A Retrospective Cohort Study

**DOI:** 10.3390/jcm14186485

**Published:** 2025-09-15

**Authors:** Alaa Atamna, Yaara Wazana, Haim Ben-Zvi, Tzippy Shochat, Jihad Bishara, Amir Nutman

**Affiliations:** 1 Infectious Diseases Unit, Beilinson Hospital, Rabin Medical Center, Petah-Tikva 491000, Israel; 2 Gray Faculty of Medical and Health Sciences, Tel-Aviv University, Tel-Aviv 6997801, Israel; 3 Clinical Microbiology Laboratory, Beilinson Hospital, Rabin Medical Center, Petah-Tikva 491000, Israel; 4 Research Authority, Beilinson Hospital, Rabin Medical Center, Petah-Tikva 491000, Israel; 5 Department of Epidemiology and Preventive Medicine, Gray Faculty of Medical and Health Sciences, Tel-Aviv University, Tel-Aviv 6997801, Israel; 6 Hospital Management, Wolfson Medical Center, Holon 58100, Israel

**Keywords:** carbapenem-resistant *Acinetobacter baumannii* (CRAB), bacteremia, mortality, early antibiotic treatment, infection outcomes, colistin

## Abstract

Carbapenem-resistant *Acinetobacter baumannii* (CRAB) bacteremia is a critical health concern associated with high morbidity and mortality and limited treatment options. Whether early initiation of concordant antibiotic therapy upon recognition of sepsis improves outcomes remains unclear. **Methods**: We conducted a retrospective cohort study of 413 patients diagnosed with CRAB bacteremia to evaluate the impact of early concordant antibiotic treatment (i.e., administration of in vitro active antibiotics within 24 h of blood culture collection) on 30-day mortality. Multivariable logistic regression was conducted to identify predictors of early concordant treatment and to evaluate its association with 30-day mortality. To address potential confounding by early death, a sensitivity analysis was performed which included only patients who survived at least 48 h after blood culture collection. **Results**: Among the study cohort, 30% (122/413) received early concordant treatment (all received colistin), while 70% (291/413) received early discordant treatment. The median age of patients receiving early concordant treatment was 69 (interquartile range (IQR), 62–78) years vs. 71 (IQR, 62–81) years in the discordant group (*p* = 0.1). Patients who received early concordant treatment were more likely to be mechanically ventilated (52% vs. 40%, *p* = 0.03) and have rectal carriage of multidrug-resistant bacteria (16% vs. 9%, *p* = 0.06). The 30-day mortality was 63% (260/413). In univariate analysis, survivors were more likely to have received early concordant treatment (38% vs. 25%, *p* = 0.005); however, this association was not statistically significant in the multivariable model (adjusted odds ratio [aOR] 0.36, 95% confidence interval [CI] 0.13–1.02, *p* = 0.053). Significant factors associated with 30-day mortality included age ≥65 years (aOR 4; 95% CI 1.1–17, *p* = 0.04) and SOFA score ≥5 points (aOR 7.14; 95% CI 2–25, *p* < 0.01). In the sensitivity analysis limited to patients who survived at least 48 h after blood culture collection, early concordant treatment remained unassociated with 30-day mortality (aOR 1.8; 95% CI 0.5–7, *p* = 0.4). **Conclusions**: Early concordant antibiotic treatment was not significantly associated with 30-day mortality in patients with CRAB bacteremia. Older age and SOFA score were significant predictors of mortality. Whether this finding reflects the limited efficacy of colistin, which was the predominant empiric antibiotic in this cohort, remains unclear; nevertheless, more effective therapeutic options for CRAB bacteremia are urgently needed to improve patient outcomes.

## 1. Introduction

*Acinetobacter baumannii* is a non-fermentative Gram-negative coco-bacilli that is considered one of the top priority pathogens according to the World Health Organization [1]. *A. baumannii* is frequently resistant to beta lactams including carbapenems, quinolones and aminoglycosides, rendering it a difficult to treat pathogen with limited therapeutic options [2,3,4,5].

Carbapenem-resistant *A. baumannii* (CRAB) is responsible for nosocomial infections such as ventilator associated pneumonia and bloodstream infections [6] and is associated with high mortality rates that can reach over 60% [7,8,9,10,11]. In one multicenter study from Italy, the 30-day mortality among 281 episodes of CRAB bacteremia was 73.6% [12]. In another case–control study from Israel, the overall 14-day mortality of CRAB bacteremia in 172 patients was 45.2% [13].

Several studies have shown that empiric microbiologically concordant antibiotic treatment for bacteremia is associated with reduced odds of mortality. In a meta-analysis of 48 studies, discordant empirical antibiotic treatment in adults with sepsis was associated with a 60% increase in all-cause mortality (odds ratio [OR], 1.6; 95% confidence interval [CI], 1.37–1.86) [14]. A recent large retrospective study of approximately 20,000 patients with bacteremia demonstrated that discordant empirical antibiotic therapy was associated with increased mortality (adjusted OR [aOR] 1.46; 95% CI 1.28–1.66) [15]. Beyond microbiological concordance, the timeliness of antibiotic administration is also critical; each hour of delay in effective therapy during the first 6 h of septic shock was associated with a 7.6% decrease in survival [16].

Studies evaluating the impact of early concordant treatment in CRAB bacteremia are scarce. The limited and often suboptimal therapeutic options, including polymyxins such as colistin, are associated with reduced efficacy and significant toxicity. As a result, delays in initiating effective therapy may contribute to poor outcomes. Kim et al. reported a high 28-day mortality rate of 69.8% among patients with CRAB bacteremia who did not receive concordant antibiotic therapy. Notably, 88.9% of deaths occurred within the first five days [17]. Nutman et al. reported a lower 14-day mortality among patients with CRAB bacteremia who received concordant antibiotic therapy within five days [13]. Another retrospective study from South Korea reported that colistin treatment initiated within 5 days and administered for more than 48 h was significantly associated with lower 28-day mortality in patients with CRAB bacteremia (OR 0.31; 95% CI 0.11–0.88) [18].

In the current study, we aimed to assess the impact of administering concordant antibiotics within the first 24 h of blood culture collection on 30-day mortality in patients with CRAB bacteremia, in order to evaluate both the adequacy and timeliness of empirical antibiotic therapy.

## 2. Methods

A retrospective cohort study that included all consecutive hospitalized patients with CRAB bacteremia in Beilinson hospital, a 900-bed tertiary teaching hospital in central Israel, between 2012 and 2022. The cohort was divided into two groups based on whether they received concordant antibiotic treatment within 24 h of blood culture collection (concordant group) or not (discordant group). Concordance was determined retrospectively, based on in vitro susceptibility of the isolated CRAB strain, regardless of whether culture results were available at the time of administration. The primary outcome was 30-day mortality. Patients with polymicrobial bacteremia were excluded.

### 2.1. Definitions

CRAB bacteremia was defined as at least one positive blood culture for CRAB (see Section 2.2), with the onset of bacteremia determined by the timing of blood culture collection.

Early concordant treatment was defined as administration of at least one antimicrobial agent with in vitro activity against the CRAB isolate within 24 h of blood culture collection.

Early discordant treatment was defined as either no antimicrobial agent administered within 24 h, or administration of only agents without in vitro activity against the CRAB isolate.

### 2.2. Microbiological Methods

Isolates of the *A. baumannii* complex were identified using the Microflex MALDI-TOF MS system with Biotyper software (Version 5.1.420.61) (Bruker Daltonics, Billerica, MA, USA). Species-level identification was performed based on Biotyper score thresholds and database matches [19].

Antimicrobial susceptibility was determined using the Kirby–Bauer disk diffusion susceptibility test. Isolates were considered carbapenem-resistant if they were non-susceptible to imipenem or meropenem.

Colistin susceptibility was determined using the MICRONAUT MIC-Strip (MERLIN Diagnostika, Bornheim, Germany). Reference minimum inhibitory concentrations (MICs) for colistin were interpreted in accordance with ISO standard 20776-1 and CLSI/EUCAST recommendations [20].

### 2.3. Data Collection

Data were collected from patients’ electronic medical records and included demographic variables (age, gender, place of residence), body mass index (BMI), comorbidities, clinical characteristics at CRAB bacteremia onset, laboratory results, and antibiotic treatment data. Comorbidities were recorded individually (including ischemic heart disease, congestive heart failure, diabetes mellitus, chronic kidney disease, solid tumors, hematologic malignancies, and solid organ or bone marrow transplantation), and the Charlson comorbidity index (CCI) was calculated for each patient to quantify baseline comorbidity burden based on diagnoses documented prior to the CRAB bacteremia episode. Clinical parameters at the time of bacteremia onset included mechanical ventilation status, presence of a central venous catheter, rectal carriage of multidrug-resistant (MDR) organisms (CRAB or carbapenem-resistant Enterobacterales (CRE)), and severity of illness as assessed by the Sequential Organ Failure Assessment (SOFA) score. Laboratory values retrieved included white blood cell count, serum creatinine, and albumin. Data on antibiotic treatments, including agents administered and their timing relative to blood culture collection, were also collected. The source of bacteremia was defined according to the CDC/NHSN criteria [21].

### 2.4. Statistical Analysis

Descriptive statistics were used to summarize demographic and clinical characteristics of individuals diagnosed with CRAB bacteremia. Categorical variables were presented as frequencies and percentages, while continuous variables were summarized based on their distribution: normally distributed variables were reported as means with standard deviations (SD), and non-normally distributed variables were reported as medians with interquartile ranges (IQR). The Kolmogorov–Smirnov test was used to assess normality.

Comparisons between groups, based on the concordance of early antibiotic treatment, were conducted using the Chi-square test or Fisher’s exact test for categorical variables. Continuous variables were compared using an independent samples *t*-test for normally distributed variables and the Wilcoxon rank-sum test for non-normally distributed variables.

Multivariable logistic regression was performed to identify predictors of early concordant antibiotic treatment and to evaluate the association between early concordant antibiotic treatment and 30-day mortality. Variables with a *p*-value < 0.1 in bivariate analysis were included in the multivariable model.

To account for potential confounding by early death—as many patients in the discordant treatment group died before antibiotic initiation—an additional analysis of 30-day mortality was performed. This analysis included only patients who survived at least 48 h following blood culture collection. The 48 h threshold was chosen because many early deaths occurred within the first few hours of sepsis onset, and because the clinical benefits of microbiologically concordant antibiotic treatment may take time to manifest.

Statistical analyses were performed using SAS 9.4 (SAS Institute Inc., Cary, NC, USA). Statistical significance was set at *p* < 0.05, and all tests were two-tailed.

## 3. Results

The study included 413 patients with CRAB bacteremia. All bacteremia were monobacterial. The median days from admission to bacteremia onset was 11 (IQR 3–20) days. Most patients with CRAB bacteremia were hospitalized in medical wards (320/413, 77%) followed by surgical wards (78/413, 19%) and intensive care units (ICUs) (13/413, 4%) (Table 1).

Of the study cohort, 122/413 (30%) received early concordant treatment—all of whom were treated with colistin—while 291/413 (70%) received early discordant treatment. All CRAB isolates had a MIC for colistin below 1 mg/L. Among those who received early discordant treatment, 201 had received an antibiotic to which the CRAB isolate was not susceptible, while 90 patients did not receive antibiotic treatment at all within 24 h. The median age of patients who received concordant treatment was 69 (IQR 62–78) years as compared to 71 (IQR, 62–81) years for patients who received discordant treatment. Patients who received early concordant treatment were more likely to have rectal carriage of CRAB or CRE (20/122, 16% vs. 26/291, 9%, *p* = 0.06) (Table 1).

At the time of CRAB bacteremia presentation, the median SOFA score was 4 in both groups (*p* = 0.2). Patients in the concordant treatment group were more likely to be mechanically ventilated (63/122, 52% vs. 115/291, 40%; *p* = 0.03). Central venous catheter use was similar between groups (67/122, 55% vs. 152/291, 52%; *p* = 0.7). Pneumonia was the most common source of bacteremia in both groups (69/122, 57% vs. 124/291, 44%, *p* = 0.1) followed by primary bacteremia (34/122, 28% vs. 114/291, 39%, *p* = 0.1) (Table 1).

Factors associated with receipt of early concordant treatment were mechanical ventilation at time of bacteremia onset (aOR 1.9; 95% CI 1.1–3.1), rectal carriage of MDR (aOR 1.9; 95% CI 1.02–3.8), and CCI score ≥3 points (aOR 2.7; 95% CI 1.3–5.5) (Table 2).

The 30-day mortality among CRAB bacteremia patients was 260/413, 63%. Risk factors for 30-day mortality are presented in Table 3. Deceased patients were older (median age 73 vs. 67 years, *p* < 0.01), had higher comorbidity burden (median CCI score 5 vs. 4, *p* = 0.05), and more frequently had congestive heart failure (70/260, 27% vs. 20/153, 13%, *p* <0.01). Furthermore, deceased patients were more likely to develop bacteremia later (≥7 days) during hospitalization (176/260, 68% vs. 88/153, 58%, *p* = 0.05), had higher SOFA scores (median 5 vs. 3, *p* < 0.01), and were more likely to require mechanical ventilation (124/260, 48% vs. 54/153, 35%, *p* = 0.01). A smaller proportion of deceased patients received early concordant therapy compared to survivors (64/260, 25% vs. 58/153, 38%, *p* = 0.005) (Table 3).

In the multivariable model, after adjusting for age ≥65 years, CCI ≥3 points, congestive heart failure, SOFA score at bacteremia onset, and timing of bacteremia onset, early concordant treatment was no longer associated with 30-day mortality (aOR = 0.36, 95% CI 0.13–1.02, *p* = 0.053), reaching only borderline statistical significance. Factors independently associated with increased mortality included age ≥65 years (aOR 4; 95% CI 1.1–17) and SOFA score ≥5 points (aOR 7.14; 95% CI 2–25) (Table 4).

To account for potential confounding by early death—where rapid clinical deterioration may have precluded initiation of antibiotics—we performed a sensitivity analysis excluding 173 patients who did not survive at least 48 h after blood culture collection. Among the remaining 240 patients, 30-day mortality occurred in 87 (36%), of whom 38 (44%) had received early concordant treatment. In comparison, only 58/153 (38%) of survivors had also received early concordant treatment (*p* = 0.4). Notably, the direction of the association was reversed compared to the primary analysis, and remained non-significant after adjustment for other variables including age, CCI, and SOFA score (aOR for 30-mortality with concordant vs. discordant treatment: 1.8; 95% CI 0.5–7).

## 4. Discussion

In this study, early concordant treatment for CRAB bacteremia did not significantly improve 30-day survival as compared with those who received early discordant treatment. Factors associated with higher 30-day mortality included older age (≥65 years) and a SOFA score ≥5. In the context of CRAB bacteremia, a higher SOFA score likely reflects greater physiologic derangement and severity of organ dysfunction at the time of infection onset, which may limit the benefit of antibiotic intervention [13,22].

Few studies have focused on the influence of concordant empiric treatment on mortality in patients with CRAB bacteremia. A retrospective study from South Korea showed that colistin treatment within 5 days and for more than 48 h was significantly associated with lower 28-day mortality in patients with CRAB bacteremia (OR = 0.31; 95% CI, 0.11–0.88) [18]. Another retrospective small study of 40 patients from Greece with *A. baumannii* bacteremia showed that patients who did not receive concordant treatment within 3 days of a taking blood culture had a higher mortality rate as compared to those who received concordant treatment (59.1% vs. 33.3%, *p* = 0.1) [23].

In contrast to previous studies that defined early treatment over broader timeframes (3–5 days), our study examined the effect of initiating concordant antibiotic therapy within the first 24 h after blood culture collection—a more stringent and clinically relevant window that reflects real-time decision-making in the management of sepsis. Importantly, 70% of patients did not receive concordant therapy within this timeframe, and a substantial proportion died before appropriate antibiotics could be administered. In the primary analysis, early concordant therapy was not significantly associated with reduced 30-day mortality (aOR 0.36, 95% CI 0.13–1.02; *p* = 0.053), despite a trend toward benefit. To address potential confounding by early death, we conducted a sensitivity analysis limited to patients who survived at least 48 h after blood culture collection (42% of the cohort). In this subgroup, early concordant therapy again showed no significant association with mortality—and notably, the direction of the association reversed (aOR 1.8, 95% CI 0.5–7.0). This reversal, although statistically non-significant, reinforces the interpretation that early concordant therapy, when limited to colistin, may not meaningfully impact mortality in this setting. These results highlight the urgent need for more effective therapies for CRAB bloodstream infections [24].

Factors associated with early concordant treatment were CCI ≥ 3 points, mechanical ventilation at time of bacteremia presentation and rectal carriage of CRAB or CRE bacteria. In one observational study from China, the incidence of subsequent CRAB infection was twice as high in patients with CRAB intestinal carriage compared to those without carriage (*p* < 0.001) [25].

Our study has several limitations. First, as a single-center study, the generalizability of our findings to other institutions may be limited. For instance, all CRAB isolates in our study had low MIC for colistin, which may reduce the applicability of our findings to centers with higher rates of colistin-resistant CRAB isolates. Second, the retrospective design makes the study susceptible to information bias. Third, the classification of concordant antibiotic treatment was based on in vitro susceptibility testing, which may not accurately reflect in vivo effectiveness due to factors such as tissue penetration, bacterial virulence, and host immune response. This is particularly relevant for colistin, which was the only agent used in the early concordant treatment group. Given its poor tissue penetration and suboptimal pharmacokinetics, our findings may primarily reflect the limited clinical effectiveness of early colistin administration rather than the broader impact of early concordant therapy. Furthermore, the susceptibility testing method used (MICRONAUT MIC-Strip) may not reliably detect hetero-resistance to colistin, potentially overestimating susceptibility and contributing to treatment failure despite in vitro concordance. Moreover, newer agents with improved activity against CRAB, such as cefiderocol and sulbactam–durlobactam, were not available during the study period. Future studies should evaluate the impact of these therapies when used empirically in the early phase of CRAB bacteremia [26]. Fourth, we did not collect data on antibiotic treatment prior to CRAB bacteremia or on the use of combination therapy, both of which may have influenced treatment selection and clinical outcomes. Fifth, information on secondary outcomes, including infection resolution, recurrence, duration of bacteremia, ICU length of stay, and need for mechanical ventilation, was not collected, limiting assessment of clinical course beyond mortality. Sixth, although we excluded patients who died within 48 h in a sensitivity analysis to address potential confounding by early mortality, some degree of residual confounding may still persist. However, given that early concordant therapy was not significantly associated with mortality in either the primary or sensitivity analyses—and that the direction of effect reversed in the latter—such bias is unlikely to have masked a true benefit, and would more plausibly have attenuated any association.

In conclusion, early concordant antibiotic treatment—administered within 24 h of blood culture collection—was not significantly associated with reduced 30-day mortality in patients with CRAB bacteremia. These findings suggest that in real-world settings where colistin is the only available empiric option, early concordant therapy may offer limited survival benefit. Improving outcomes in this high-risk population will require faster diagnostics, better risk stratification, and access to more effective empiric agents beyond colistin.

## Figures and Tables

**Table 1:** Characteristics of hospitalized patients with CRAB bacteremia according to whether they received concordant treatment * within 24 h.

	Concordant Treatment (N = 122)	Discordant Treatment (N = 291)	*p*-Value
Baseline characteristics			
Age (years), median (IQR)	69 (62–78)	71 (62–81)	0.1
Age ≥ 65 years, n (%)	82 (67%)	200 (69%)	0.8
Female, n (%)	49 (40%)	119 (41%)	0.9
CCI, median (IQR)	4 (2–6)	4 (3–7)	0.07
CCI ≥ 3 points, n (%)	89 (73%)	236 (81%)	0.08
Home residency, n (%)	53 (43%)	133 (46%)	0.9
Body mass index (kg/m^2^), median (IQR)	26 (23–30)	26 (23–30)	0.9
Prior hospitalization within 3 months, n (%)	69 (57%)	143 (49%)	0.2
Ischemic heart disease, n (%)	19 (16%)	54 (19%)	0.6
Congestive heart failure, n (%)	26 (21%)	64 (22%)	1
Diabetes mellitus, n (%)	28 (23%)	77 (26%)	0.5
Chronic kidney disease, n (%)	3 (2%)	8 (3%)	1
Solid tumor, n (%)	20 (16%)	51 (18%)	0.9
Hematological malignancy, n (%)	13 (11%)	27 (9%)	0.7
Bone marrow transplantation, n (%)	6 (5%)	6 (2%)	0.2
Solid organ transplantation, n (%)	11 (9%)	12 (4%)	0.06
Characteristics at bacteremia onset			
SOFA score, median (IQR)	4 (2–5)	4 (3–7)	0.2
SOFA score ≥ 5 points, n (%)	14 (11%)	32 (11%)	0.8
Mechanical ventilation, n (%)	63 (52%)	115 (40%)	0.03
Central venous catheter, n (%)	67 (55%)	152 (52%)	0.7
CRAB or CRE carriage, n (%)	20 (16%)	26 (9%)	0.06
Late bacteremia onset (≥7 days of admission), n (%)	82 (67%)	182 (63%)	0.4
White blood cell count (×10^3^ cells/µL), median (IQR)	10 (6–16)	9 (6–15)	0.5
Creatinine level (mg/dL), median (IQR)	1.2 (0.7–1.9)	1.2 (0.8–2)	0.3
Albumin level (g/dL), median (IQR)	3 (2.6–3.5)	3 (2.5–3.5)	0.9
Albumin level ≤ 3 g/dL, n (%)	62 (51%)	159 (55%)	0.6
Bacteremia source			
Pneumonia, n (%)	69 (57%)	127 (44%)	0.1
Urinary tract, n (%)	4 (3%)	8 (3%)	0.1
CLABSI, n (%)	10 (8%)	18 (6%)	0.1
Intraabdominal, n (%)	3 (2%)	10 (3%)	0.1
Surgical site infection, n (%)	2 (2%)	14 (5%)	0.1
Primary bacteremia, n (%)	34 (28%)	114 (39%)	0.1
Outcome			
30 days mortality, n (%)	64 (52%)	196 (67%)	0.04

IQR—interquartile range; CCI—Charlson comorbidity index; SOFA—sequential organ failure assessment; CRAB—carbapenem-resistant *Acinetobacter baumannii*; CRE—carbapenem-resistant Enterobacterales; CLABSI—central line-associated bloodstream infections. * Administration of at least one antimicrobial agent with in vitro activity against the CRAB isolate.

**Table 2:** Multivariable analysis of predictors for early concordant treatment * for CRAB bacteremia.

Variable	Adjusted OR (95% CI)	*p*-Value
Age ≥ 65 vs. <65 years	1.3 (0.7–2.5)	0.4
Mechanical ventilation at time of bacteremia Yes vs. No	1.9 (1.1–3.1)	0.02
Late bacteremia presentation (≥7 days) vs. earlier presentation (<7 days)	1.1 (0.6–1.9)	0.8
CCI ≥3 vs. <3 points	2.7 (1.3–5.5)	<0.01
Rectal carriage of CRAB or CRE Yes vs. No	1.9 (1.02–3.8)	0.04
Prior hospitalization within 3 months Yes vs. No	1.5 (0.9–2.6)	0.1

OR—odds ratio; CI—confidence interval; CCI—Charlson comorbidity index; CRAB—carbapenem-resistant *Acinetobacter baumannii*; CRE—carbapenem-resistant Enterobacterales. * Administration of at least one antimicrobial agent with in vitro activity against the CRAB isolate.

**Table 3:** Univariate analysis of risk factors for 30-day mortality in hospitalized patients with CRAB bacteremia.

Variable	Survived (N = 153)	Deceased (N = 260)	*p*-Value
Baseline characteristics			
Age (years), median (IQR)	67 (51–76)	73 (65–81)	<0.01
Age ≥ 65 years, n (%)	82 (54%)	200 (78%)	<0.01
Female gender, n (%)	57 (37%)	111 (43%)	0.3
CCI, median (IQR)	4 (2–7)	5 (3–7)	0.05
CCI ≥ 3 points, n (%)	106 (69%)	219 (84%)	<0.01
Body mass index (kg/m^2^), median (IQR)	25 (23–29)	26 (23–30)	0.8
Home residency, n (%)	65 (42%)	121 (47%)	0.4
Prior hospitalization within 3 months, n (%)	76 (50%)	136 (52%)	0.6
Ischemic heart disease, n (%)	22 (14%)	51 (20%)	0.2
Congestive heart failure, n (%)	20 (13%)	70 (27%)	<0.01
Diabetes mellitus, n (%)	31 (20%)	74 (28%)	0.07
Chronic kidney disease, n (%)	6 (4%)	5 (2%)	0.2
Solid tumor, n (%)	30 (20%)	41 (16%)	0.3
Hematological cancer, n (%)	10 (7%)	30 (12%)	0.1
Bone marrow transplantation, n (%)	2 (1%)	10 (4%)	0.2
Solid organ transplantation, n (%)	12 (8%)	11 (4%)	0.1
Clinical characteristics at time of bacteremia onset			
SOFA score, median (IQR)	3 (2–5)	5 (4–8)	<0.01
SOFA score ≥ 5 points, n (%)	11 (7%)	35 (13%)	<0.01
Mechanical ventilation, n (%)	54 (35%)	124 (48%)	0.01
Central venous catheter, n (%)	85 (56%)	134 (52%)	0.4
Late bacteremia onset (≥7 days of admission), n (%)	88 (58%)	176 (68%)	0.05
CRAB or CRE carriage, n (%)	19 (12%)	27 (10%)	0.5
CRAB pneumonia, n (%)	74 (48%)	122 (47%)	0.7
Primary bacteremia, n (%)	47 (31%)	101 (39)	0.1
White blood cell count (×10^3^ cells/µL), median (IQR)	10 (7–16)	9 (6–14)	0.5
Creatinine level (mg/dL), median (IQR)	0.9 (0.7–1.7)	1.4 (0.8–2)	0.3
Albumin level (g/dL), median (IQR)	3.1 (2.6–3.5)	3 (2.5–3.5)	0.2
Albumin level ≤ 3 g/dL, n (%)	68 (44%)	118 (45%)	0.7
Early concordant treatment *, n (%)	58 (38%)	64 (25%)	0.005

IQR—interquartile range; CCI—Charlson comorbidity index; SOFA—sequential organ failure assessment; CRAB—carbapenem-resistant *Acinetobacter baumannii*; CRE—carbapenem-resistant Enterobacterales. * Administration of at least one antimicrobial agent with in vitro activity against the CRAB isolate.

**Table 4:** Multivariable analysis of risk factors for 30-day mortality.

Variable	Adjusted OR (95% CI)	*p*-Value
Early concordant treatment * vs. discordant treatment	0.36 (0.13–1.02)	0.053
Age ≥ 65 vs. <65 years	4 (1.1–17)	0.04
CCI ≥ 3 vs. <3 points	2.5 (0.5–14.3)	0.3
Mechanical ventilation Yes vs. No	1.6 (0.57–4.6)	0.4
Congestive heart failure Yes vs. No	1.2 (0.3–5)	0.8
Late bacteremia presentation (≥7 days) vs. earlier presentation (<7 days)	3.33 (0.77–16.66)	0.09
SOFA score ≥ 5 vs. <5	7.14 (2–25)	<0.01

OR—odds ratio; CI—confidence interval; CCI—Charlson comorbidity index; SOFA—sequential organ failure assessment. * Administration of at least one antimicrobial agent with in vitro activity against the CRAB isolate.

## Data Availability

The data presented in this study are available on request from the corresponding author.
